# Anisotropic and Coherent Control of Radical Pairs by Optimized RF Fields

**DOI:** 10.3390/ijms24119700

**Published:** 2023-06-02

**Authors:** Akihiro Tateno, Kenta Masuzawa, Hiroki Nagashima, Kiminori Maeda

**Affiliations:** Department of Chemistry, Graduate School of Science and Engineering, Saitama University, 255 Shimo-okubo, Sakura-ku, Saitama 338-8570, Japan

**Keywords:** radical pairs, local optimization, radio wave, reaction control

## Abstract

Radical pair kinetics is determined by the coherent and incoherent spin dynamics of spin pair and spin-selective chemical reactions. In a previous paper, reaction control and nuclear spin state selection by designed radiofrequency (RF) magnetic resonance was proposed. Here, we present two novel types of reaction control calculated by the local optimization method. One is anisotropic reaction control and the other is coherent path control. In both cases, the weighting parameters for the target states play an important role in the optimizing of the RF field. In the anisotropic control of radical pairs, the weighting parameters play an important role in the selection of the sub-ensemble. In coherent control, one can set the parameters for the intermediate states, and it is possible to specify the path to reach a final state by adjusting the weighting parameters. The global optimization of the weighting parameters for coherent control has been studied. These manifest calculations show the possibility of controlling the chemical reactions of radical pair intermediates in different ways.

## 1. Introduction

A radical pair (RP) is composed of two radicals, each of which has an unpaired electron. When RPs are produced photochemically, the initial electron spin state of the RPs preserves the spin manifold of the precursor excited state. Although this fact has been known for a long time, it has been re-evaluated and has attracted much attention because this state is the so-called “entanglement state” [1,2,3,4,5]. On the other hand, the formation of RPs is also known in photoreceptor protein systems, such as photosynthetic reaction centers [6,7,8,9] and cryptochromes (CRYs) [10,11,12,13,14]. In particular, CRYs are candidate molecules for magnetoreception in animals, and the relationship between magnetism and biological systems has been discussed [15,16,17,18,19]. The influence of not only magnetic fields but also that of electromagnetic waves has been discussed [20]. In the geomagnetic perception of migratory birds, the influence of electromagnetic noise is still under discussion [21].

**Figure 1:**
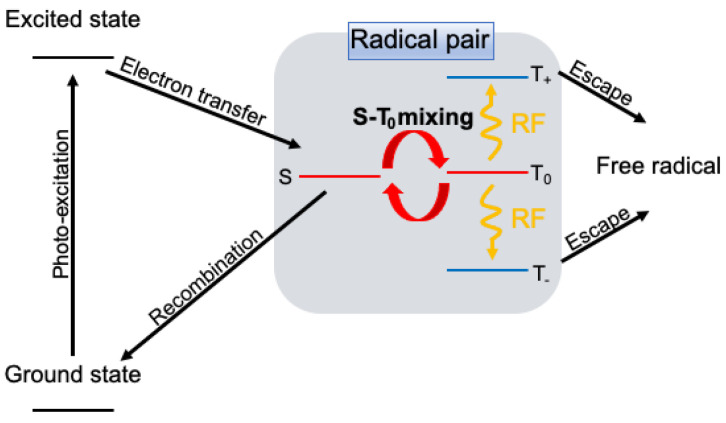
Reaction scheme for the formation of singlet RPs, and reactivity control by RF transitions (RYDMR).

The most probable effect of electromagnetic waves on RPs is magnetic resonance [22]. It is called reaction yield detected magnetic resonance (RYDMR) [23,24,25,26,27,28,29] and is measured at different external magnetic fields and resonance frequencies. Figure 1 shows a schematic diagram of the spin state and chemical reactivity of RPs. Here, we consider the typical case where a singlet-born radical pair reacts spin-selectively from the singlet state under the magnetic field. At the high magnetic field, the microwave frequency matching with the Zeeman splitting energy induces the resonance phenomena, producing $T_{+}$ and $T_{-}$ states, which do not have singlet properties and are retained for a long time or become free radicals by radical diffusion. Therefore, recombination reactions from the singlet state are significantly suppressed by the magnetic resonance phenomena. On the other hand, in a small magnetic field at the geomagnetic level, Zeeman splitting is smaller than hyperfine coupling, so the resonance of the radiofrequency to hyperfine coupling is important. Such magnetic resonance phenomena of RPs are complex, but the resulting change in the singlet character of RPs affects the reaction yield. In contrast to Figure 1, the spin states of general RPs are more complex. The resonance frequency of RPs is the same as the electron spin resonance frequency of the two constituent radicals when the interaction between the radicals is negligibly small. However, they are complicatedly split by hyperfine coupling and its anisotropy. If high-field approximation holds and mixing between the nuclear spin states is small, these resonance lines can be considered as sub-ensembles. In such a case, the selection of these sub-ensembles can be conducted by selecting the RF frequency.

The spin states of a radical pair can be regarded as a quantum mechanical system. Therefore, the control of chemical reactions associated with the spin manipulation of RPs is said to be a quantum state manipulation. In the field of magnetic resonance, an optimization technique called GRAPE (Gradient Ascent Pulse Engineering) [30] is widely used. GRAPE optimizes the waveform globally on the time axis, but it tends to be trapped in local optimal states due to the combination of many segments of the RF field. In this sense, it is not a perfect global optimization for multiple variables. Therefore, in many cases, it is necessary to set many waveforms as initial conditions to find the global optimal waveform, which inevitably increases the computational complexity. Therefore, the application of this method is limited to a small number of short pulse designs and is difficult to optimize the RF field for the chemical reaction control to occur over long periods of time.

On the other hand, a local optimization method with respect to the time axis has been used to design an infrared laser field for vibronic state control [31]. This method works very well for relatively simple quantum states and the optimal wave can be obtained in a very short computation time. In contrast to the GRAPE method, this method requires essentially no iterative computation. Thus, the optimized waveform and the resulting time evolution of the system are obtained simultaneously in a single calculation. The method is based on the concept of “living for the day” and monotonically increases fidelity, i.e., the overlap of the system with the target state. In fact, although it is a very crude method, it shows excellent results for quantum systems achieved with relatively simple paths.

We have applied this local optimization method to control the chemical reaction of RPs. This method has the following features:

(1) Smooth waveforms can be obtained because waveforms can be computed with very high time resolution. In contrast, the waveforms computed in GRAPE are optimized for relatively coarse time steps.
(2) Sub-ensemble systems can be easily selected by resonance frequency in inhomogeneous RP systems.
(3) The calculation can incorporate strategies for the time evolution of the quantum system with respect to its path.
(4) Calculations in a rotational coordinate system are easy because there is no computational burden due to the multidimensionality of radio waves (microwaves).

In particular, sub-ensemble selection (2) and path control in coherent control (3) can be achieved by using the same sets of parameters, called weighting parameters {$q_{i}$}, which will be discussed in the next section. In a previous paper [32], we demonstrated (2) nuclear spin state selections under (3) high-field approximation. In the present paper, we present anisotropic reaction control and coherent control in an extremely low magnetic field, focusing on the weighting parameters. Furthermore, we have improved the local-optimization-based coherent control of the spin system of RPs via the global optimization of a few weighting parameters. Of course, this method is not perfect global optimization, but it is a significant improvement. The model system used here is rather simple compared to realistic systems. However, once the methodology is developed and a strategy for reaction control is established, it should be easy to apply them to a realistic system because the computational burden is significantly lower.

## 2. Theory

We have previously applied the local optimization theory, which was used for the control of vibronic systems using an infrared laser [31], to the design of RF magnetic field for the control of RP chemical reactions [32]. In order to extend computational flexibility and to take into account spin-selective chemical reactions and spin relaxations, the basic theory of the local optimization theory has been reformulated in Liouville space. In this chapter, we provide an overview of the theory and describe the important weighting parameters in control. Then, anisotropic control and coherent pathway control are formulated as an example of control using weighting parameters.

### 2.1. Local Optimization Theory

We begin with an overview of the local optimization theory for our reaction control [31,32]. In the local optimization theory, the degree of achievement of a desired quantum state is set as a performance index, and control is realized by designing an external oscillating field such that the performance index increases monotonically. Defining ${|P\rangle }_{\;}$ as the projection operator of the target quantum state (where ${|P\rangle }_{\;}$ considers eigenstates of system Hamiltonian but can be extended to non-eigenstates), the performance index y(t) is described as follows.

(1) y(t)=〈P|ρ(t)〉

|ρ(t)〉 is the density matrix of the system, and its time evolution follows the Liouville von Neuman equation in Liouville space.

(2) $$\frac{d{|\rho \left(t\right)\rangle }_{\;}}{dt}=-i\left[{\hat{\hat{L}}}_{0}+u\left(t\right){\hat{\hat{L}}}_{V}\right]{|\rho \left(t\right)\rangle }_{\;}$$

where ${\hat{\hat{L}}}_{0}+u\left(t\right){\hat{\hat{L}}}_{V}$ is composed of the static term, ${\hat{\hat{L}}}_{0}$, which drives the time evolution by the static Hamiltonian $\hat{H}$, and time dependent term, $u\left(t\right){\hat{\hat{L}}}_{V}$, which represents the interaction with the optimized oscillating RF fields u(t):

(3) $${\hat{\hat{L}}}_{0}^{\;}=\hat{H}-\tilde{H},\;{\hat{\hat{L}}}_{V}=\frac{g_{A}\mu_{B}}{\hbar }\left({\hat{S}}_{x}^{A}-{\tilde{S}}_{x}^{A}\right)+\frac{g_{B}\mu_{B}}{\hbar }\left({\hat{S}}_{x}^{B}-{\tilde{S}}_{x}^{B}\right)$$

where hat and tilde denote the operation to ket and bra in Hilbert spaces, respectively.

From ${\hat{\hat{L}}}_{0}^{\;}{|P\rangle }_{\;}=0$, the time derivative of the performance index y(t) is written by:

(4) $$\frac{dy\left(t\right)}{dt}=-iu\left(t\right)\langle P|{\hat{\hat{L}}}_{V}{|\rho \left(t\right)\rangle }_{\;}$$

Now, we introduce an amplitude parameter A, which determines the degree of optimization as well as the weighting parameter, which will be discussed later, and if u(t) is given by

(5) $$u\left(t\right)=iA\langle P|{\hat{\hat{L}}}_{V}|\rho \left(t\right)\rangle \;\left(A>0\right)$$

a monotonical increase in y(t) is guaranteed.

(6) $$\frac{dy\left(t\right)}{dt}=A{\left[\langle P|{\hat{\hat{L}}}_{V}|\rho \left(t\right)\rangle \right]}^{2}\geq 0$$

Substituting Equation (5) into Equation (2), we obtain the nonlinear differential equation shown in Equation (7).

(7) $$\frac{d{|\rho \left(t\right)\rangle }_{\;}}{dt}=-i\left[{\hat{\hat{L}}}_{0}+iA{\hat{\hat{L}}}_{V}\langle P|{\hat{\hat{L}}}_{V}|\rho \left(t\right)\rangle \right]|\rho \left(t\right)\rangle$$

Once we solve the time evolution of |ρ(t)〉, we can obtain u(t) by Equation (5).

### 2.2. Weighting Parameters

Equation (1) was an evaluation function of control targeting a single quantum state, but to target multiple quantum states, we extend Equation (1) as follows.

(8) $$y\left(t\right)=\underset{i}{\sum }q_{i}\langle P_{i}|\rho \left(t\right)\rangle$$

Here, we introduce the weighting parameters $q_{i}$, which determine the priority to the target states ${|P_{i}\rangle }_{\;}$. They are useful for the following two types of control.

One is sub-ensemble control, in which each sub-ensemble, such as different nuclear spin configurations under a high field approximation, isotope selection, and any inhomogeneous spectral broadening of the spin systems. Here, we focus on the orientation of an anisotropic system with respect to laboratory coordinates. By setting weighting parameters for each sub-ensemble, it is possible to induce ($q_{i}>0$) or suppress ($q_{i}<0$) transitions only in certain sub-ensembles.

The other is path control. By setting the weighting parameters $q_{1}<q_{2}<\cdots <q_{N}$ for the quantum states $|P_{1}\rangle ,|P_{2}\rangle ,\cdots ,|P_{N}\rangle$, transitions can occur in the order $|P_{1}\rangle \to |P_{2}\rangle \to \cdots \to |P_{N}\rangle$. In other words, these parameters can be thought of as guiding the transition path to produce a particular spin state at a particular time. From the next chapter, we will formulate these controls in a radical pair system.

### 2.3. Anisotropic Sub-Ensemble Control

Here, we consider RPs with an anisotropic hyperfine interaction. The sub-ensemble can be determined by the orientation with respect to the laboratory coordinates. The Hamiltonian of the anisotropic hyperfine interaction is described as follows.

(9) $${\hat{H}}_{HFC}=\hat{S}\cdot {\hat{A}}_{arb}\cdot \hat{I}$$

The operators $\hat{S}$ and $\hat{I}$ correspond to the electron and nucleus spin operators, respectively, and ${\hat{A}}_{arb}$ is a hyperfine tensor. ${\hat{A}}_{arb}$ is diagonalized by multiplying the appropriate rotation matrix:

(10) $$\hat{A}=\left(\begin{array}{ccc} A_{x_{M}x_{M}} & 0 & 0\\ 0 & A_{y_{M}y_{M}} & 0\\ 0 & 0 & A_{z_{M}z_{M}} \end{array}\right)$$

**Figure 2:**
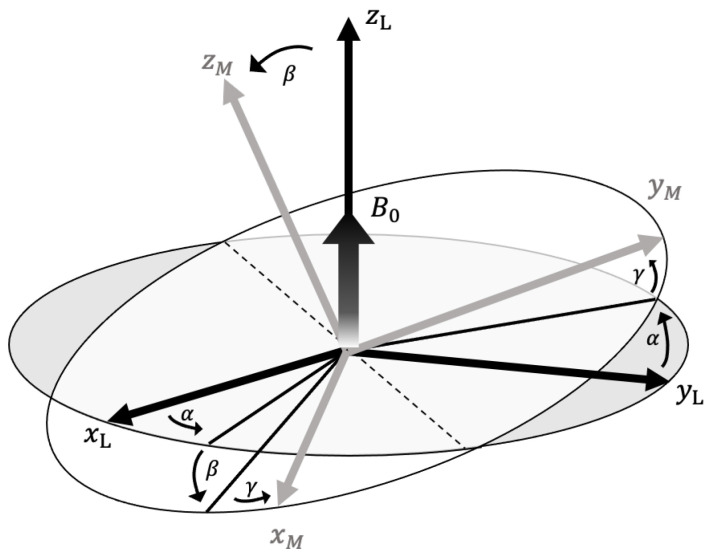
The relationship between molecular frame ($x_{M},\;y_{M},\;z_{M}$) and laboratory frame ($x_{L},\;y_{L},\;z_{L}$) defined by Euler angles (α, β, γ). $B_{0}$ is defined to be parallel to $z_{L}$.

The subscripts $x_{M},\;y_{M},\;z_{M}$ correspond to x, y, z in the molecular coordinate system. To describe the anisotropic response of a molecule to the direction of a static magnetic field $B_{0}$, the molecular coordinate system needs to be transformed into a laboratory frame. The orientation of these two coordinates systems is defined by the following Euler angles as shown in Figure 2. The conversion of the coordinates can be described by a rotational matrix R(α, β, γ):

(11) $$R\left(\alpha ,\beta ,\gamma \right)=\left(\begin{array}{ccc} \cos\gamma \cos\beta \cos\alpha -\sin\gamma \sin\alpha & \cos\gamma \cos\beta \sin\alpha +\sin\gamma \cos\alpha & -\cos\gamma \sin\beta \\ -\sin\gamma \cos\beta \cos\alpha -\cos\gamma \sin\alpha & -\sin\gamma \cos\beta \sin\alpha +\cos\gamma \cos\alpha & \sin\gamma \sin\beta \\ \sin\beta \cos\alpha & \sin\beta \sin\alpha & \cos\beta \end{array}\right)$$

By using R(α, β, γ), the hyperfine tensor in laboratory frame ${\hat{A}}_{L}$ can be calculated by:

(12) $${\hat{A}}_{L}\left(\alpha ,\beta ,\gamma \right)=R\left(\alpha ,\beta ,\gamma \right)\;\hat{A}\;R^{-1}\left(\alpha ,\beta ,\gamma \right)$$

Here, we introduce the new index k={α, β, γ} for the sub-ensembles of RPs. The Hamiltonian of a radical pair with one nuclear spin (I=1/2) for a sub-ensemble k is given by:

(13) $${\hat{H}}_{L}^{k}=\frac{g_{A}\mu_{B}B_{0}}{\hbar }{\hat{S}}_{z}^{A}+\frac{g_{B}\mu_{B}B_{0}}{\hbar }{\hat{S}}_{z}^{B}+\frac{g_{N}\mu_{N}B_{0}}{\hbar }{\hat{I}}_{z}^{A}+{\hat{S}}^{A}\cdot {\hat{A}}_{L}\left(k\right)\cdot {\hat{I}}^{A}-J\left(\frac{1}{2}+2{\hat{S}}^{A}\cdot {\hat{S}}^{B}\right)$$

The first and second terms correspond to the electron spin Zeeman interaction of radical A and radical B, the third term indicates the nuclear Zeeman interaction, the fourth term denotes the hyperfine interaction, and the fifth term represents the exchange interaction of the RPs.

Since the index k represents the sub-ensemble of orientations relative to the magnetic field direction of the system, the total density vector ${|\rho \rangle }_{\;}$ can be divided into the vector ${|\rho_{k}\rangle }_{\;}$ for each sub-ensemble k. Thus, the performance index y(t) can be defined by:

(14) $$\begin{array}{ll} y\left(t\right) & =\int_{k}^{\;}q_{k}\langle P{}_{k}|\rho_{k}{\left(t\right)\rangle }_{\;}\left|\sin\beta \right|dk\\ & =\underset{k}{\sum }q_{k}^{j}\langle P{}_{k}|\rho_{k}{\left(t\right)\rangle }_{\;} \end{array}$$

$P_{k}$ is the projection operator for the target states, and $q_{k}$ is the weighting factor for $P_{k}$. $q_{k}^{j}$ is the product of the weighting factor $q_{k}$ and the Jacobian |sinβ| ($q_{k}^{j}=q_{k}\times \left|\sin\beta \right|$). The $P_{k}$ can be constructed as follows:

1. The eigenstates of ${\hat{H}}_{L}^{k}$ were calculated.
2. The two eigenstates with the largest $T_{+\alpha }$ and $T_{-\beta }$ characteristics were chosen, where the state $T_{+\alpha }$ and $T_{-\beta }$ indicate $T_{+}$ electron spin state with α nuclear spin and $T_{-}$ with β nuclear spin, respectively.
3. The sum of the projection operators onto these two eigenstates was determined to be the target, $P_{k}$.

The magnetic resonance transitions from the singlet rich eigenstates to the target steady states $P_{k}$ increase y(t) and provide long-lived RP sub-ensembles. By an appropriate setting of the weighting parameters, $q_{k}$ one can select the specific sub-ensembles and transfer the population to the stable eigenstates that are free from the recombination reaction.

The kinetics of the RPs reaction of each sub-ensemble k can be calculated by the Haberkorn super operator, ${\hat{\hat{L}}}_{K},$ using the projection operator ${\hat{P}}_{S}=\left|S\rangle \langle S\right|\;$ [33,34,35].

(15) $${\hat{\hat{L}}}_{K}=\frac{-ik_{S}}{2}\left({\hat{P}}_{S}+{\tilde{P}}_{S}\right)$$

Since $\left({\hat{\hat{L}}}_{0}^{k}+{\hat{\hat{L}}}_{K}\right){|P\rangle }_{L}\neq 0$, occasionally y(t) decreases by the recombination kinetics. Therefore, this calculation is limited to the condition where $k_{S}$ is much smaller than the frequency of spin dynamics. In this model calculation, we have set $k_{S}=1\times 10^{6}\;s^{-1}$, such that the introduction of recombination reactions has a minimal effect on the optimized waveforms and does not break down the calculation.

The calculation process of anisotropic reaction control is performed in the same way as the previous calculation, i.e., solving the Liouville von Neuman equation, Equation (17) using optimized u(t), which guarantees a monotonous increase in y(t):

(16) $$u\left(t\right)=iA\underset{k}{\sum }q_{k}^{j}\langle P_{k}|{\hat{\hat{L}}}_{V}|\rho_{k}{\left(t\right)\rangle }_{\;}\;\left(A>0\right)$$

(17) $$\frac{d{|\rho_{k}\left(t\right)\rangle }_{\;}}{dt}=-i\left[{\hat{\hat{L}}}_{0}^{k}+{\hat{\hat{L}}}_{K}+iA{\hat{\hat{L}}}_{V}\underset{k}{\sum }q_{k}^{j}\langle P_{k}|{\hat{\hat{L}}}_{V}|\rho_{k}\left(t\right)\rangle \right]{|\rho_{k}\left(t\right)\rangle }_{\;}$$

### 2.4. Coherent Pathway Control

In coherent control, the non-eigenstate of the Hamiltonian is set as the target state. Since the non-eigenstate evolves automatically in time, the target state ${|f\rangle }_{\;}$ should be set at a specific time $t_{f}$, and reverse time evolution should be introduced.

(18) $${|\rho_{f}\rangle }_{\;}=exp\left\{i{\hat{\hat{L}}}_{0}\left(t_{f}-t\right)\right\}{|f\rangle }_{\;}$$

where ${\hat{\hat{L}}}_{0}$ is a Liouvillian of field-free Hamiltonian ${\hat{H}}_{0}$. We call this time-dependent target state ${|\rho_{f}\rangle }_{\;}$ the moving target. If the density vector |ρ〉 of the system can be reached to the moving target by applying the external field at time t, the desired state ${|f\rangle }_{\;}$ can be obtained automatically at time $t_{f}$. In a magnetic field, the singlet state becomes an unsteady state due to S-T_0_ mixing. Therefore, the singlet state is a candidate for the target of coherent control. If the singlet state can be maximized at $t_{f}$, a spin-selective reaction, such as a recombination reaction, can be promoted. However, depending on the target state, it may be difficult to directly reach the moving target. Even in such a case, the moving target can be obtained by introducing the intermediate state |i〉 and the weighting factor q [36].

(19) $${|\rho_{i\to f}\rangle }_{\;}=exp\left\{i{\hat{\hat{L}}}_{0}\left(t_{f}-t\right)\right\}\left[q_{i}{|i\rangle }_{\;}+q_{f}|f\rangle \right]$$

Here, the roles of $q_{i},\;q_{f}$ are not the same as $\;q_{k}\;$ in Section 2.3, but are rather parameters for specifying the transition path. By setting $q_{f}>q_{i}$, it becomes possible to specify a transition path that transitions to the state ${|f\rangle }_{\;}$ at time $t_{f}$ via the state |i〉. In such a way, one can input strategies into the spin dynamics of RPs.

To evaluate the degree of achievement of $|\rho_{i\to f}\rangle$, the performance index y(t) is defined as:

(20) $$y\left(t\right)=\langle \rho_{i\to f}|\rho \left(t\right)\rangle$$

The time evolution of ${|\rho \left(t\right)\rangle }_{\;}$ is described by the Liouville von Neuman equation, so the time derivative of y(t) is given by:

(21) $$\begin{array}{ll} \frac{dy\left(t\right)}{dt} & =\left[q_{i}\langle i|+q_{f}\langle f|\right]i{\hat{\hat{L}}}_{0}exp\left\{i{\hat{\hat{L}}}_{0}\left(t-t_{f}\right)\right\}{|\rho \left(t\right)\rangle }_{\;}+\langle \rho_{i\to f}|\frac{d{|\rho \left(t\right)\rangle }_{\;}}{dt}\\ & =i\langle \rho_{i\to f}|{\hat{\hat{L}}}_{0}{|\rho \left(t\right)\rangle }_{\;}-i\langle \rho_{i\to f}|\left[{\hat{\hat{L}}}_{0}+{\hat{\hat{L}}}_{V}u\left(t\right)\right]{|\rho \left(t\right)\rangle }_{\;}\\ & =-iu\left(t\right)\langle \rho_{i\to f}|{\hat{\hat{L}}}_{V}{|\rho \left(t\right)\rangle }_{\;} \end{array}$$

As in previous discussions, by defining u(t) as Equation (22), a monotonous increase in Equation (20) is guaranteed (Equation (23)).

(22) $$u\left(t\right)=iA\langle \rho_{i\to f}|{\hat{\hat{L}}}_{V}{|\rho \left(t\right)\rangle }_{\;}\;\left(A>0\right)$$

(23) $$\frac{dy\left(t\right)}{dt}=A{\left[\langle \rho_{i\to f}|{\hat{\hat{L}}}_{V}|\rho \left(t\right)\rangle \right]}^{2}\geq 0$$

As a result, the equation that the coherent controlled system follows is following a nonlinear differential equation.

(24) $$\frac{d{|\rho \left(t\right)\rangle }_{\;}}{dt}=-i\left[{\hat{\hat{L}}}_{0}+iA{\hat{\hat{L}}}_{V}\langle \rho_{i\to f}|{\hat{\hat{L}}}_{V}|\rho \left(t\right)\rangle \;\right]|\rho \left(t\right)\rangle$$

In the coherent control calculations, we assume a single nuclear spin as in the anisotropy calculations. However, we consider only isotropic hyperfine coupling. The spin Hamiltonian is given by:

(25) $${\hat{H}}_{0}=\frac{g_{A}\mu_{B}B_{0}}{\hbar }{\hat{S}}_{z}^{A}+\frac{g_{B}\mu_{B}B_{0}}{\hbar }{\hat{S}}_{z}^{B}+\frac{g_{N}\mu_{N}B_{0}}{\hbar }{\hat{I}}_{z}^{A}+a_{A}\;{\hat{S}}^{A}\cdot {\hat{I}}^{A}-J\left(\frac{1}{2}+2{\hat{S}}^{A}\cdot {\hat{S}}^{B}\right)$$

In addition, for coherent control, we did not consider the recombination reaction.

### 2.5. Parameter Global Optimization

The parameters used for control, such as the weighting parameter $q_{i}$. and the amplitude parameter A, have a strong influence on the fidelity of the desired quantum state |f〉 depending on their values. To obtain the desired state with high fidelity, it is necessary to use an appropriate combination of parameters. Therefore, we define the fidelity of the target state obtained by the one control as a function of $q_{i}$ and A, Equation (26), and use global optimization (ga function in MATLAB Global Optimization Toolbox [37,38,39]) to find the combination of parameters that maximizes the fidelity of |f〉.

(26) $$f\left(q_{i},\;A\right)=\langle f|\rho \left(t_{f}\right)\rangle$$

While global optimization requires iterative computations and is computationally expensive, it is more likely to obtain the desired parameters without being trapped by a local minimum due to its holistic search. In contrast, computations based on the local optimization theory do not require iterative computations, so the one-time computational cost is very low and practical control results can be obtained, but whether the obtained results are the best results cannot be determined in a single computation. Therefore, a global search of the parameters necessary for local optimization is a good method that combines the advantages of both local and global optimization since it can calculate optimal values while reducing the computational cost of each iteration of the search. Using this method, the optimal parameters for coherent control were obtained ($q_{S},\;q_{T_{\pm }},q_{T_{+\alpha },\;T_{-\beta }},\;A$ described below, which was obtained by this method).

## 3. Results and Discussion

### 3.1. Anisotropic Reaction Control

Model RPs g-factors are set as $g_{A}=2.000,\;g_{B}=2.002$, and external magnetic field $B_{0}$ was fixed to 3.6 mT. The following anisotropic hyperfine tensor was used.

(27) $$\hat{A}\left(/\mathrm{mT}\right)=\left(\begin{array}{ccc} 1.2 & 0 & 0\\ 0 & 1.2 & 0\\ 0 & 0 & 1.8\; \end{array}\right)$$

The exchange interaction J was fixed to 0.1 MHz. The initial state of RP is assumed to be singlet state.

**Figure 3:**
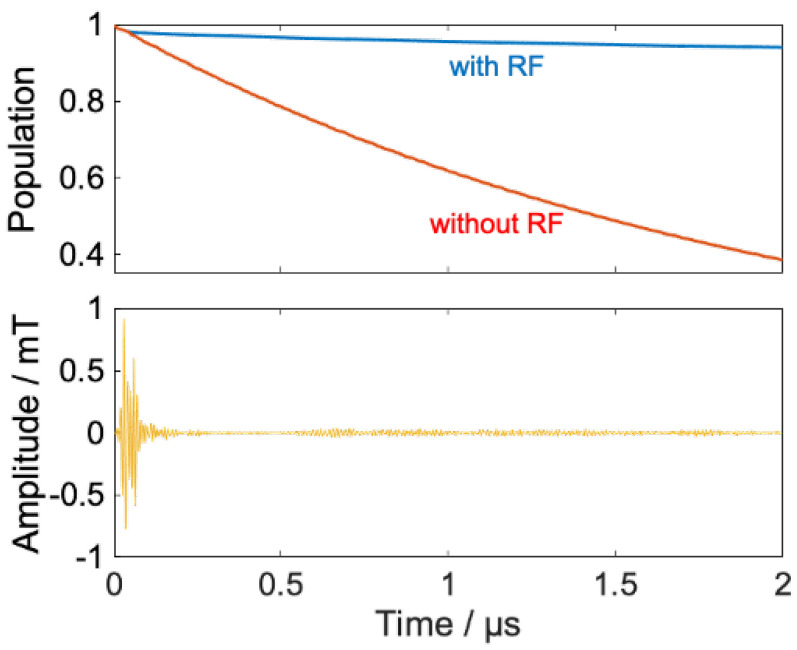
(**Upper**) Time evolution of model RP’s population (blue line: with RF, red line: without RF). (**Lower**) calculated RF field.

First, we designed a waveform that provides a transfer of the radical pair from S-T_0_ mixed states to $P_{k}$ states homogeneously. This control corresponds with keeping the RP population isotropic in the magnetic field direction by setting $q_{k}=1$ for all k. Figure 3 shows the results of the optimized RF field and decay of RPs with and without RF.

Figure 3 shows that 2 μs after radical pair formation, the population of RPs, which is 38% in the case of no RF irradiation, is successfully maintained at 94% by RF irradiation. This is a result of the RF-induced transition of the radical pair from the singlet to states with high $T_{+\alpha }$ and $T_{-\beta }$ character, which are hardly involved in S-T_0_ mixing, and the suppression of the recombination reaction.

**Figure 4:**
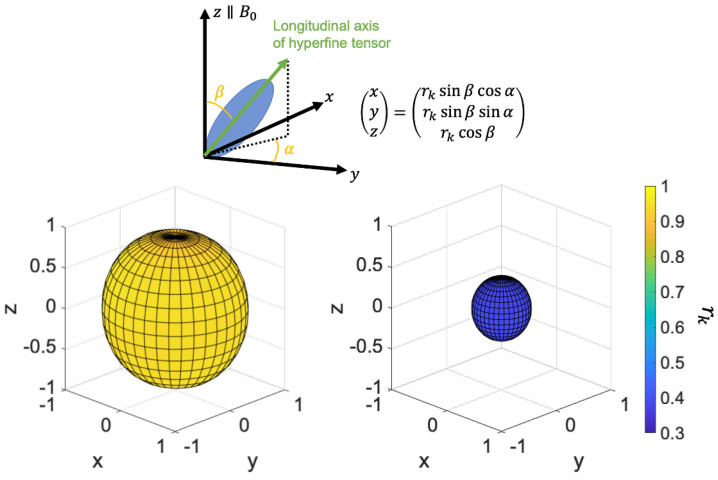
(**Upper**) Correspondence between the long axis direction of the hyperfine tensor and the coordinates (x, y, z). The length and color of $r_{k}$ represents RP’s population. Notation of angles is consistent with Figure 2. (**Lower**) RP population (*t* = 2 μs) with RF (**left**), without RF (**right**).

We now examine the anisotropy of the RP population using the coordinates shown in the top panel of Figure 4. Figure 4 shows the RP population at t = 2 μs during RF irradiation and non-irradiation, indicating that the RP population is maintained isotropically during RF irradiation.

**Figure 5:**
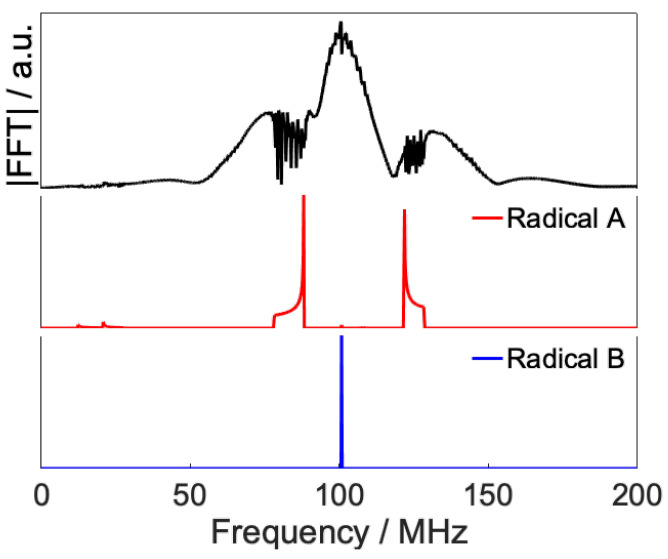
Fourier transform of calculated radio waveform (Figure 3) and simulation results of frequency swept powder ESR spectrum of the model RP calculated by an Easy Spin package (Pepper) with minimum line width.

Figure 5 shows the results of a Fourier transform of the irradiated RF signals, and it can be confirmed that the frequency components corresponding to the transitions of radical B are dominant. This is because the transition to radical B, which has isotropic interactions, is advantageous for maintaining an isotropic population.

From now on, we consider the control of the RP population anisotropic with respect to the external static magnetic field direction ($z_{L}$). At first, we tried to keep the RPs with nearly parallel orientation with respect to the external field, i.e., $z_{L}∥z_{M}$. We set $q_{k}=5$ for β = 0~50°, 130~180°, and $q_{k}=-3$ for β = 60~120°. The negative value directly means a penalty for the undesired transition.

**Figure 6:**
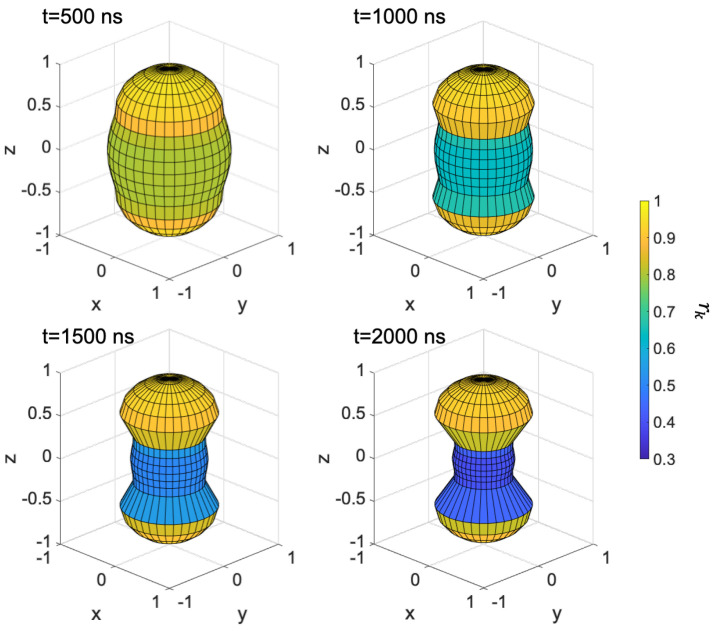
Time evolution of the anisotropic control to retain when the RPs whose longitudinal axis of hyperfine tensor is nearly parallel to the magnetic field is held.

Figure 6 shows the results of the anisotropic control. The RP population decays by recombination for sub-ensembles β= 60~120° and the component, for β = 0~50°, 130~180° stayed longer as RPs. This is because the RF-induced transitions to the eigenstates with relatively high $T_{+\alpha }$ and $T_{-\beta }$ character for the sub-ensemble β = 0~50°, 130~180° remarkably suppress the recombination efficiency, while the recombination was not suppressed for the sub-ensemble of β = 60~120° because the transitions were not induced.

**Figure 7:**
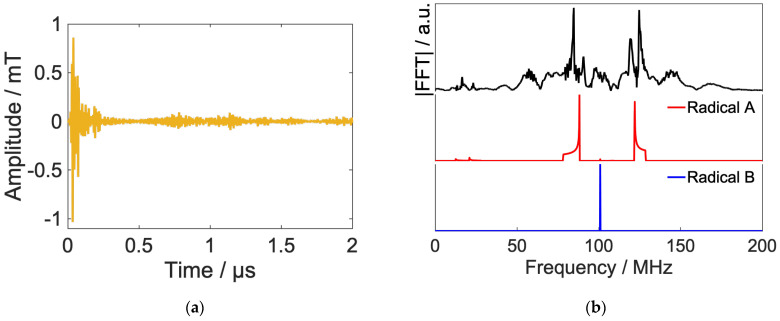
(**a**) Optimized RF field for anisotropic control. (**b**) Fourier transform of (**a**) and simulated frequency swept powder ESR spectrum of model RP.

The Fourier transform of the optimized waveform is shown in Figure 7. In contrast to the isotropic control case, the algorithm for the optimized waveform has switched the target radical transition from radical B to radical A, because radical A has the frequency distribution of the transition due to the anisotropic hyperfine interaction.

Next, we tried to keep the RPs of the β = 60~120° sub-ensemble longer. For this, the time weighting parameters were set to $q_{k}=-2.5$ for β = 0~50°, 130~180° sub-ensemble and $q_{k}=5$ for the β = 60~120° sub-ensemble.

**Figure 8:**
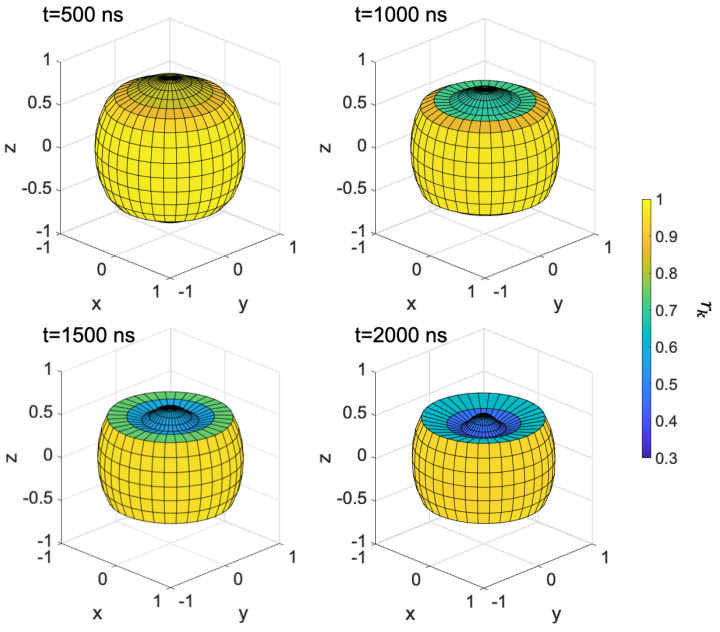
Time evolution of the anisotropic control to retain RPs whose longitudinal axis of hyperfine tensor is relatively perpendicular to the magnetic field.

**Figure 9:**
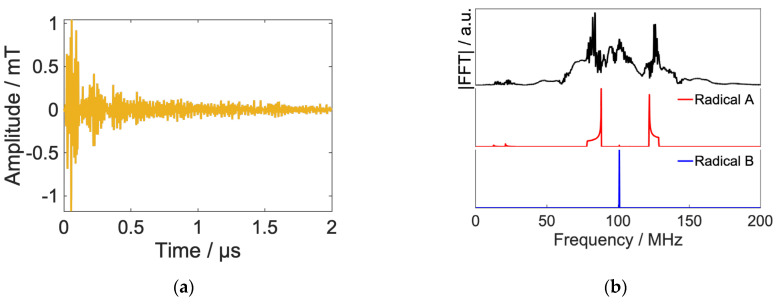
(**a**) Optimized RF field. (**b**) Fourier transform of (**a**) and simulation results of frequency swept powder ESR spectrum of model RP.

**Figure 10:**
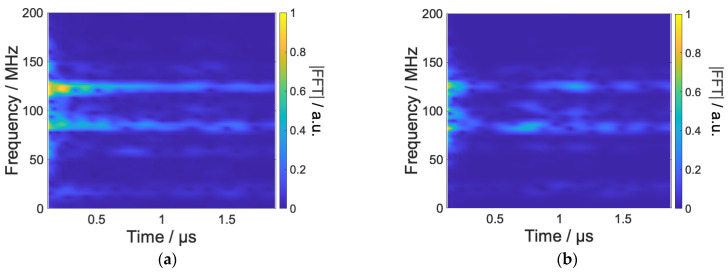
(**a**) Short-time Fourier-transformed time-resolved spectra of the optimized waveform in Figure 7, (**b**) that of waveform in Figure 9. The Hanning window of which the length and the hop size are 256 ns and 16 ns, respectively.

In contrast to the previous result, RPs whose hyperfine tensors are relatively oriented perpendicular to the magnetic field direction are retained, while RPs whose hyperfine tensors are oriented parallel to the magnetic field direction are reduced, as shown in Figure 8. Figure 9 shows the optimized waveform and the result of the Fourier transform. The frequency components corresponding to the transitions of radical A tend to dominate, but the components corresponding to the transitions of radical B are increased compared to the previous control. In order to confirm the timing of the frequency components used in the control, we performed short-time Fourier transform, as shown in Figure 10.

Figure 10 shows that the frequency components of radical A are used for a long time in both the controls shown in Figure 6 and Figure 8, while the frequency components of radical B are used only in the early time (see around 100 MHz). In the early stage, the transition to radical B contributes to the total RPs being maintained for a longer time, thus contributing to the increase in the performance index y(t). On the other hand, in the later part, isotropic transitions to radical B are avoided in order to avoid an increase in the sub-ensemble with penalized weighting parameters.

Our calculation can increase the performance index by selecting the RF frequency precisely over a long period of time, ~2 μs. In practice, however, most of the control is achieved relatively early. In the three examples shown in Figure 3, Figure 7 and Figure 9, the performance index *y*(*t*) at *t* = 0.25 μs is 97%, 79%, and 92% of *y*(*t*) at *t* = 2 μs, respectively.

### 3.2. Coherent Pathway Control

Now, we discuss coherent control. For coherent control, the hyperfine coupling constant $a_{A}$ was set to 15 MHz (≈0.54 mT), the RP’s g-factors were set as $g_{A},\;g_{B}=2.000$, and the exchange interaction J was set to 0.1 MHz. Under this condition, only the hyperfine interaction contributes to S-T_0_ mixing. The calculation was performed under external magnetic fields of 20 mT and 0.05 mT. As in the previous section, the initial state of the RP was set to the singlet state, and the calculation was performed so that the RP returned to the singlet state again after 1  μs via the T_±_ state. To handle this control, the moving target was set as Equation (28).

(28) $${|\rho_{T_{\pm }\;\to S}\rangle }_{\;}=exp\left\{i{\hat{\hat{L}}}_{0}\left(t_{f}-t\right)\right\}[q_{T_{\pm }\;}{|T_{\pm }\rangle }_{\;}+q_{S}|S\rangle ]$$

**Figure 11:**
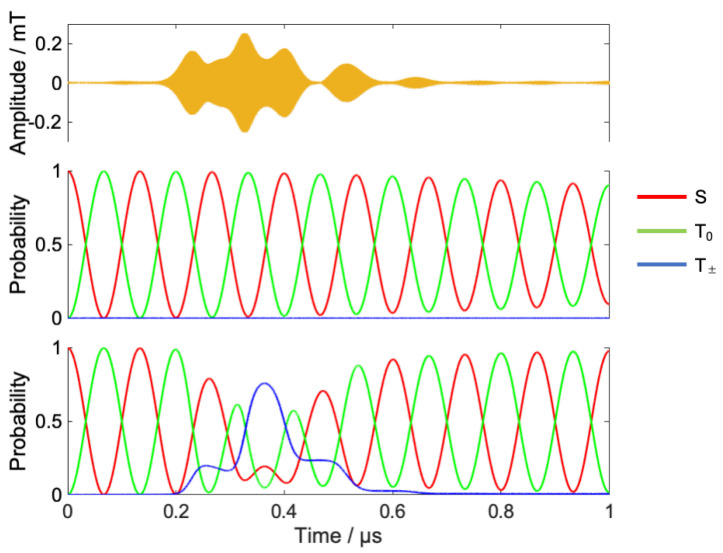
Results of the coherent control under 20 mT. Optimized RF (**upper**), time evolution of spin state without RF (**middle**), and with RF (**lower**).

Figure 11 shows the result of coherent control under $B_{0}=20\;\mathrm{mT}$.

In the high-field condition, the time evolution does not produce the probability of T_±_. Therefore, the strategy of coherent control is as follows.

1. S-T_0_ mixing increases the population of T_0_ states;
2. The RF provides a transition from the T_0_ state to the T_±_ state;
3. The transition from T_±_ to T_0_ is again triggered by the RF, timed to maximize the singlet at the target time.

The population of the singlet state after 1 μs without RF irradiation is less than 10% due to S-T_0_ mixing. Optimized RF induces T_0_ – T_±_ transitions around 0.2  μs, and the subsequent T_±_ − T_0_ transition shifts the phase of S-T_0_ mixing, eventually returning about 98% of the RP to the singlet state at 1 μs.

**Figure 12:**
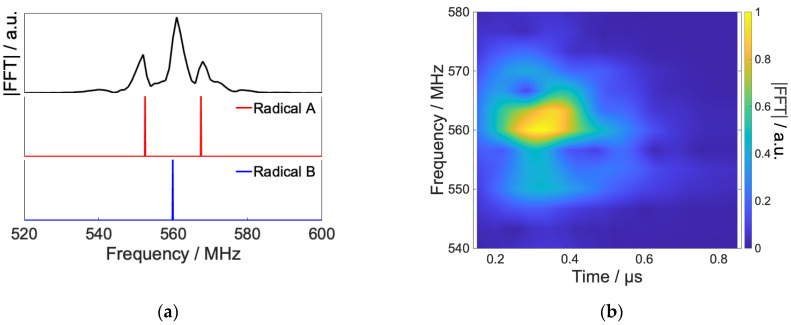
Spectrum of the calculated RF field by Fourier transform and simulation results of frequency swept ESR spectrum of model RP (**a**) and short-time Fourier transform of radio waves in Figure 11 (**b**). The Hanning window is used as the window function, and the length of the window function is 300 ns with a hop size of 10 ns.

Figure 12a,b show the power spectrum of the optimized RF and short-time Fourier transform, respectively. The optimized RF is mainly composed of radical B, which has no hyperfine interaction. The peak of this frequency component rises from 0.2 μs and decays around 0.6 μs, which is in good agreement with the time evolution of the spin state. From these results, we can confirm that the local control theory has attained the desired control by causing transitions to the radical B step by step in the control at a high magnetic field.

**Figure 13:**
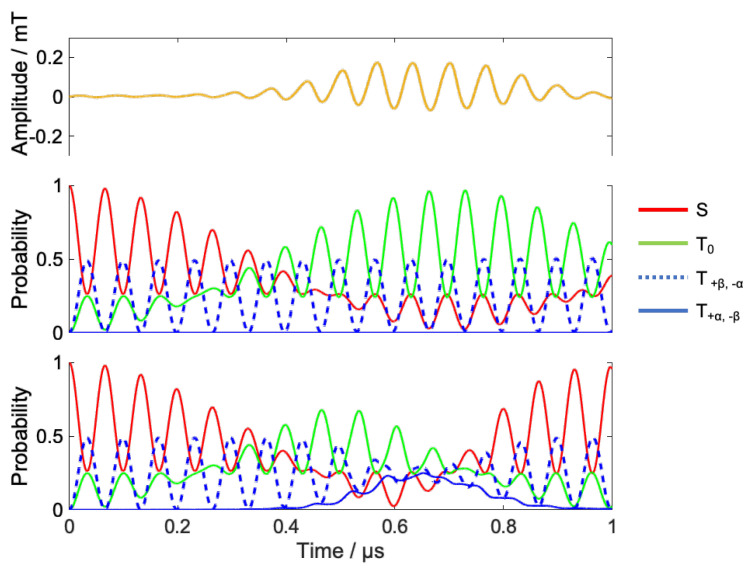
Results of the coherent control under 0.05 mT. Optimized RF (**upper**), time evolution of spin state without RF (**middle**), and with RF (**lower**).

In the extremely low magnetic field condition ($B_{0}$ = 0.05 mT), the energy separation Zeeman splitting becomes small, and hyperfine interactions dominate. As a result, not only S-T_0_ mixing but also other state mixings occur, as shown in Figure 13. The mixing between spin states looks more complicated than at 20 mT. In the model system, singlet states mix with $T_{+\beta ,-\alpha \;}$, but $T_{+\alpha ,-\beta \;}$ states remain isolated even in a low magnetic field. Only the RF field can make transitions to the isolated $T_{+\alpha ,-\beta \;}$. Therefore, these states are useful as the intermediate quantum states for the coherent control of the RPs. To achieve more efficient population control, the moving target with weighting factors for $T_{+\alpha ,-\beta \;}$ was employed.

(29) $${|\rho_{T\;\to S}\rangle }_{\;}=exp\left\{i{\hat{\hat{L}}}_{0}\left(t_{f}-t\right)\right\}\left[q_{T_{+\alpha },\;T_{-\beta }}|T_{+\alpha ,-\beta \;}\rangle +q_{S}{|S\rangle }_{\;}\right]$$

Figure 13 shows the result of coherent control under $B_{0}=0.05\;\mathrm{mT}$. By optimized RF, RP’s population were stored to $T_{+\alpha ,-\beta \;}$ states for 0.4 μs to 0.9 μs. Due to the transitions, the phase of S-T_0_ mixing was changed around 0.5 μs, and singlet fidelity was improved from 39% to 96%.

**Figure 14:**
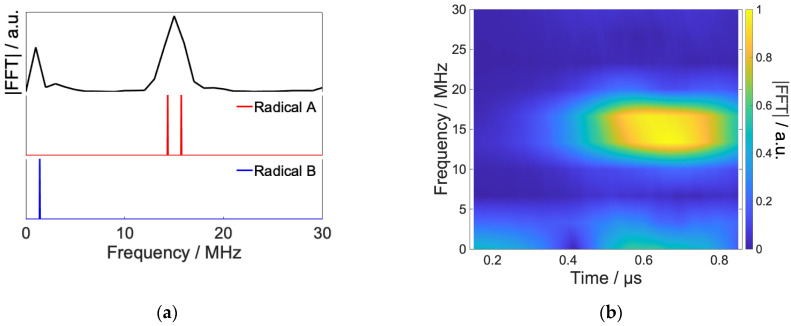
Results of Fourier transform and simulation results of frequency swept ESR spectrum of model RP (**a**) and short-time Fourier transform of radio waves in Figure 13 (**b**). Short-time Fourier transform was performed under the same conditions as in Figure 11.

Figure 14 shows the power spectrum and the short-time Fourier transform of optimized RF shown Figure 13. In contrast to the high field case, the frequency component corresponding to the transition of radical A mainly constitutes optimized RF. This is considered because the Zeeman splitting under the geomagnetic field is so small that the RF prioritizes the control of transitions between states split by nuclear spins. Thus, under very low magnetic fields such as the geomagnetic field, the broad frequency component due to the hyperfine interaction of the system can affect the reaction rather than the Zeeman splitting of the external magnetic field [21], and the present control results suggest this.

### 3.3. Parameter Optimization

**Figure 15:**
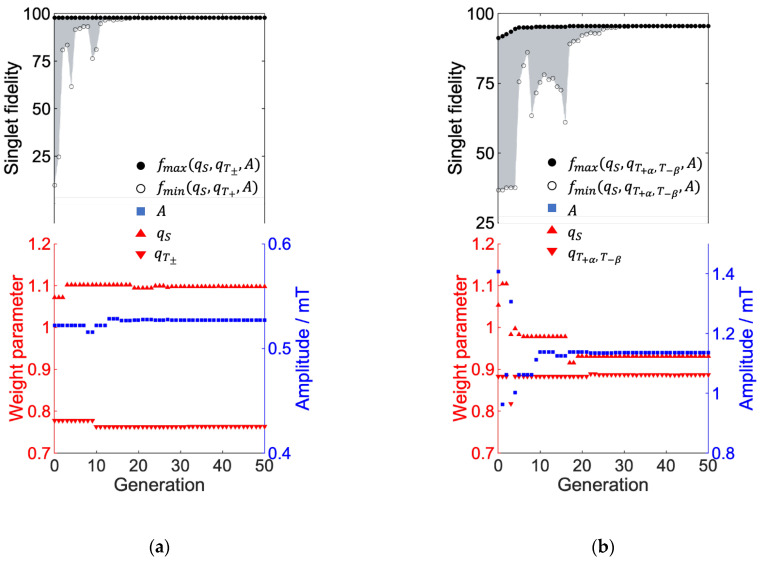
Fidelity of singlet of coherent control and evolution of each parameter value when optimized with ga function of global optimization ((**a**): high field control of Figure 11, (**b**): geomagnetic field control of Figure 13). Generation indicates the number of iterations in Global optimization, where 50 parameter combinations are computed in one generation. The parameters search range was set to $\left[0.5<q_{T_{+\alpha },\;T_{-\beta }}<0.9,\;0.9<q_{S}<1.5,\;0.1\;\left(\mathrm{mT}\right)<A<3\;\left(\mathrm{mT}\right)\right]$. $f_{max}$ and $f_{min}$ indicate highest and lowest value of the singlet yield in each generation, and the singlet fidelity values for each generation are distributed in the gray area.

Figure 15 shows the evolution of the weighting parameters $\left\{q_{i}\right\}$ and amplitude parameter *A* and Singlet fidelity at the target time (1 μs) during global optimization.

At generation zero, we set arbitrary weighting parameters and amplitude parameters. The genetic algorithm (ga function) of the MATLAB global optimization toolbox generated fifty sets of parameters {$q_{S},\;q_{T_{\pm }},\;A\;\mathrm{or}\;q_{S},\;q_{T_{+\alpha },\;T_{-\beta }},\;A$} and calculated the locally optimized RF field. In fifty sets of the calculations, the maximum and minimum fidelities, $f_{max}$ and $f_{min}$, were plotted as shown in Figure 15. This process was repeated fifty times and we can confirm the convergence of the fidelities with respect to the parameter sets. As shown in Figure 15, the number of generations required for the convergence of the parameter sets is greater for the low-field control (Figure 15b) than for the high-field control (Figure 15a). The high field control is a simple problem of how to make a phase shift of S-T_0_ mixing oscillating by a single frequency. In contrast, the control at a low magnetic field is more difficult because the coherent oscillations contain both the hyperfine coupling and the external magnetic field, and the value of the control parameter has a large effect on the fidelity of the singlet. Therefore, the high-field results converged early, while the low-field results required more generations to converge the parameters. Here, we define $\left[P_{max}\right]$ as the parameter combination $\left\{q_{S},\;q_{T_{+\alpha },\;T_{-\beta }},\;A\right\}=\left\{0.93,\;0.89,\;1.1\right\}$*,* which provides the maximum value of singlet fidelity and $\left[P_{min}\right]\;$as the parameter combination $\left\{q_{S},\;q_{T_{+\alpha },\;T_{-\beta }},\;A\right\}=\left\{0.91,\;0.80,\;0.46\right\}$, which provides the minimum value of singlet fidelity in low-magnetic-field control.

**Figure 16:**
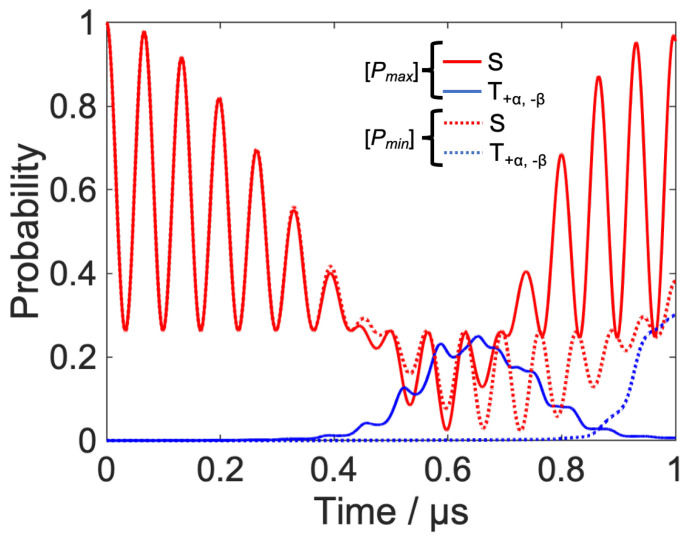
Time evolution of spin systems under low field control when $\left[P_{max}\right]$ and $\left[P_{min}\right]$ (see the text for details) are used as parameters. (solid line: control result using $\left[P_{max}\right]$, dotted line: control result using $\left[P_{min}\right]$).

Figure 16 shows the time evolution of the spin system when $\left[P_{max}\right]$ and $\left[P_{min}\right]$ are used as control parameters. The transition to the $T_{+\alpha ,-\beta \;}$ state is slower in the control using $\left[P_{min}\right]$ compared to the control using $\left[P_{max}\right]$, and the target time is reached before the RP populations return to singlet state again. This reflects the importance of the optimal combination of parameters, especially when coherent mixing is significant. Therefore, the global search for local optimization parameters used in this study is a method that can compensate for the weakness of the local optimization method and obtain better control results.

## 4. Concluding Remarks

In this paper, we focused on the weighting parameters used to control local optimization and demonstrated sub-ensemble and coherent control for a model system of RPs. By using appropriate weighting parameters, we achieved control over the selection of anisotropic sub-ensembles and the design of transition paths, albeit in a simple system.

The anisotropic response of RPs to magnetic fields is of interest from the perspective of avian magnetic compasses, and the possibility of oscillating magnetic fields affecting their dynamics is related to test experiments on animal behavior. The anisotropic induction of RP by RF irradiation presented in this paper, together with the fact that broadband RF irradiation disrupts the orientation of migratory birds [15,20,21], implies that RF irradiation of a certain waveform may be able to induce bird orientation. However, in practice, it would be difficult to apply this method to animal experiments in geomagnetic fields.

Coherent control reveals the RF waveform that maximizes the singlet state at a specific time. When singlet recombination is slow, this coherent control has little effect on the actual reaction yield. However, it has recently been reported that the coherent mixing of RPs has been directly observed by pump-push spectroscopy [40]. In this technique, the spin-selective chemical reaction is enhanced by a push laser pulse instantaneously. Therefore, we could reflect the coherent spin control into the reaction kinetics in an efficient way. The combination of this method with the coherent control by AWG-RYDMR could open up a new methodology for chemical reaction control. This calculation and the experimental verification are in progress.

Although our method uses an almost completely known spin Hamiltonian to control the chemistry of radical pairs, an inverse problem is the possibility of searching for spin Hamiltonians from this AWG-based control of chemical reactions. The intention is to optimize the AWG signal by experimental feedback and, inversely, to obtain information about the radical pairs. However, it is challenging to obtain information in this way that cannot be obtained by conventional EPR spectroscopy. Rather, such a method may be useful for obtaining kinetic information, such as recombination reactivity, or when several radical pairs with different reactivities are mixed.
